# Direct optical activation of skeletal muscle fibres efficiently controls muscle contraction and attenuates denervation atrophy

**DOI:** 10.1038/ncomms9506

**Published:** 2015-10-13

**Authors:** Philippe Magown, Basavaraj Shettar, Ying Zhang, Victor F. Rafuse

**Affiliations:** 1 Department of Medical Neurosciences, Brain Repair Centre, Life Science Research Institute, Dalhousie University, 1348 Summer Street, 3rd Floor, Halifax, Nova Scotia, Canada B3H 1X5; 2 Department of Surgery (Neurosurgery), Queen Elizabeth II Health Sciences Centre, Dalhousie University, 1796 Summer Street, 3rd Floor, Halifax, Nova Scotia, Canada B3H 3A7; 3 Department of Medicine (Neurology), Queen Elizabeth II Health Sciences Centre, Dalhousie University, 1796 Summer Street, 3rd Floor, Halifax, Nova Scotia, Canada B3H 3A7

## Abstract

Neural prostheses can restore meaningful function to paralysed muscles by electrically stimulating innervating motor axons, but fail when muscles are completely denervated, as seen in amyotrophic lateral sclerosis, or after a peripheral nerve or spinal cord injury. Here we show that channelrhodopsin-2 is expressed within the sarcolemma and T-tubules of skeletal muscle fibres in transgenic mice. This expression pattern allows for optical control of muscle contraction with comparable forces to nerve stimulation. Force can be controlled by varying light pulse intensity, duration or frequency. Light-stimulated muscle fibres depolarize proportionally to light intensity and duration. Denervated triceps surae muscles transcutaneously stimulated optically on a daily basis for 10 days show a significant attenuation in atrophy resulting in significantly greater contractile forces compared with chronically denervated muscles. Together, this study shows that channelrhodopsin-2/H134R can be used to restore function to permanently denervated muscles and reduce pathophysiological changes associated with denervation pathologies.

## Abstract (web summary)

Nerve damage can lead to skeletal muscle paralysis and atrophy. Here, the authors show that localized photostimulation of mouse calf muscle expressing the light-sensitive channel Channelrhodopsin-2 generates contraction in the absence of neural impulses and prove that this strategy can be used to prevent muscle atrophy.

## Graphical abstract

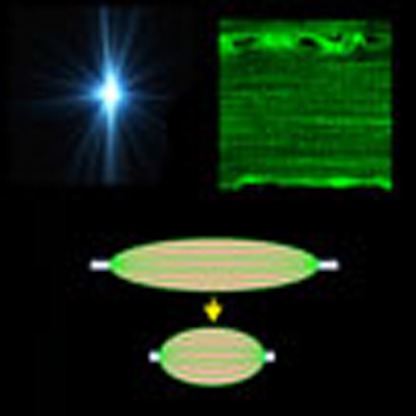

Spinal cord injuries (SCIs) and peripheral nerve injuries, such as a brachial plexus avulsion, cause severe motor deficits that ultimately impact the physical, psychological and social well-being of those affected. Restoring meaningful function to specific muscle groups, such as those controlling hand grip, can increase independence and improve overall quality of life^1^. Restoration of function to paralysed hand muscles has been achieved by combining functional electrical stimulation (FES) with neural prostheses by electrically stimulating the innervating motor nerve^2^,^3^. Neural prostheses do not function if the targeted muscles are completely denervated. Unfortunately, SCIs and peripheral nerve injuries often result in complete and permanent muscle denervation, because their associated motor neurons either died from the injury^4^ or failed to restore innervation in time. The only means to restore function to permanently denervated muscles is through cell replacement therapy (that is, motor neuron transplantations) or by a different form of exogenous activation.

Motor neurons derived from embryonic ventral cord cells^5^, embryonic stem (ES) cells^6^ or induced pluripotent stem cells^7^ have been transplanted into the peripheral nerve environment near the motor nerve entry point of completely denervated muscles. In all cases, transplanted motor neurons restored some motor functions by reinnervating muscle fibres. Furthermore, when electrically stimulated, the transplanted neurons evoked appreciable force contractions (up to 50% of control values). Recently, Bryson *et al*.^8^ extended on these studies by generating genetically modified ES-cell-derived motor neurons expressing the light-sensitive ion channel, channelrhodopsin-2 (ChR2)^9^. Optical stimulation of the transplanted ChR2 motor neurons generated contractile forces equal to 12% of control values^8^.

Studies such as these support the development of strategies to restore function to denervated muscles by combining FES technology with the transplantation of motor neurons derived from pluripotent cells. However, two portentous issues preclude introducing this technology clinically. First, several studies reviewed by Knoepfler have shown that teratomas can form from residual pluripotent cells in the transplanted population even when directed to differentiate before transplantation^10^. Second, it is well established that transected nerves and denervated muscles become refractory to growth and reinnervation over time^11^,^12^,^13^. Thus, unless motor neurons are grafted shortly after an irreversible denervation injury, functional motor recovery will likely remain poor.

To evade these issues, we tested whether direct expression of ChR2 in skeletal muscle enables efficient optical control of muscle force and function, and whether denervated muscles can be chronically activated after complete nerve transection. Optical control of muscle contraction was achieved by varying blue light pulse intensity, pulse duration and pulse frequency and generated comparable forces to neural-evoked contractions. Intracellular potentials were investigated to determine the properties of ChR2-induced depolarizations with, and without, action potential propagation along the sarcolemma. We found that muscle fibre depolarization by ChR2 activation was a function of light intensity and duration. In the presence of myosin inhibitors, a single action potential was generated, regardless of light pulse duration, followed by a rebound and plateau. Finally, we found that muscle fibre atrophy was attenuated and contractile force partially spared when denervated muscles were transcutaneously light activated for 1 h per day. Our proof-of-principle study demonstrates the capacity for fine optical control of functional muscle contraction and sparing of muscle atrophy after denervation.

## Results

### Distribution of ChR2 in Sim1-Ai32 mouse muscle

Mice expressing Cre recombinase under the control of *Sim1* regulatory sequences were bred with mice harbouring a *loxP*-flanked STOP cassette preventing the transcription of a downstream ChR2(H134R)–EYFP fusion gene (known as Ai32 mice). *Sim1* is highly expressed in skeletal muscle early in development^14^, while the ChR2/enhanced yellow fluorescent protein (EYFP) fusion protein harbours a gain-of-function mutation (H134R)^15^ in the ChR2 protein allowing larger currents^15^,^16^, greater light sensitivity and less desensitization^15^,^17^.

**Figure 1:**
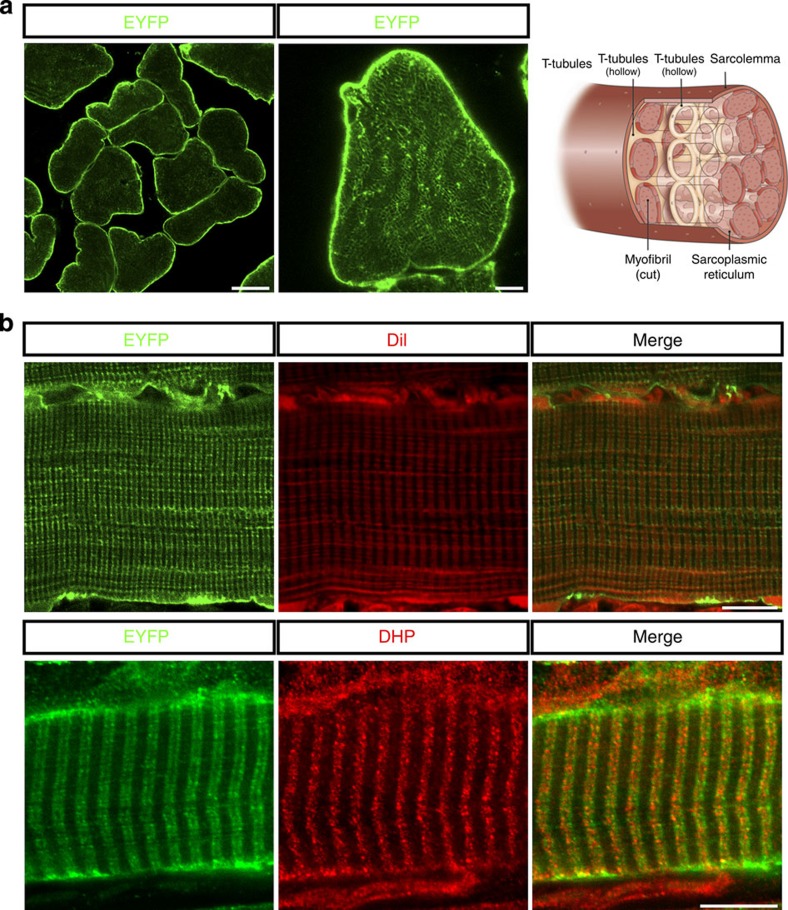
ChR2 expression in Sim1-Ai32 mouse muscle fibres. (**a**) Confocal image shows ChR2/YEFP fusion protein is highly expressed in sarcolemma (left panel, scale 20 μm) and lightly expressed in honeycomb structures within individual myofibres (middle panel). Illustration (right panel) shows key anatomical structures associated with myofibres including the sarcolemma, myofibrils and T-tubules. (**b**) Confocal image of a Sim1-Ai32 muscle fibre longitudinal section labelled with DiI (top panel) or DHP calcium channels (bottom panel) to show all membrane-associated structures including T-tubules. DiI and DHP calcium channel staining both co-localize with the EYFP/ChR2 fusion protein, indicating that ChR2 is expressed in the sarcolemma and T-tubule network. Scale bars, 5 μm (**a**) and 10 μm (**b**).

We first set out to ascertain the cellular localization of the ChR2/EYFP fusion protein in individual myofibres by immunolabelling soleus muscle cross-sections, taken from young Sim1-Ai32 mice, for EYFP. Confocal microscopy showed EYFP fluorescence was concentrated at the sarcolemma of each muscle fibre (Fig. 1a, left panel). Closer examination showed fainter EYFP immunolabelling of honeycomb structures (Fig. 1a, middle panel) resembling T-tubules within individual muscle fibres (Fig. 1a, schematic). To ascertain whether ChR2–EYFP was localized to the T-tubule network, we labelled all plasma membrane-associated structures with the lipophilic carbocyanine dye 1,1-dioctadecyl-3,3,3,3-tetramethylinocarbocyanine perchlorate (DiI)^18^. Localization of EYFP (Fig. 1b, left panel) and DiI (Fig. 1b, middle panel) along the sarcolemma and T-tubules was identified in longitudinal sections of soleus myofibres (Fig. 1b, right panel). Further confirmation of EYFP localization to the T-tubules was confirmed with co-localization with dihydropyridine (DHP) calcium channels (Fig. 1b, lower panel). Taken together, these results indicate that ChR2 is distributed throughout structures associated with muscle depolarization and excitation–contraction (EC) coupling.

### Light pulses cause muscle contraction in Sim1-Ai32 mice

**Figure 2:**
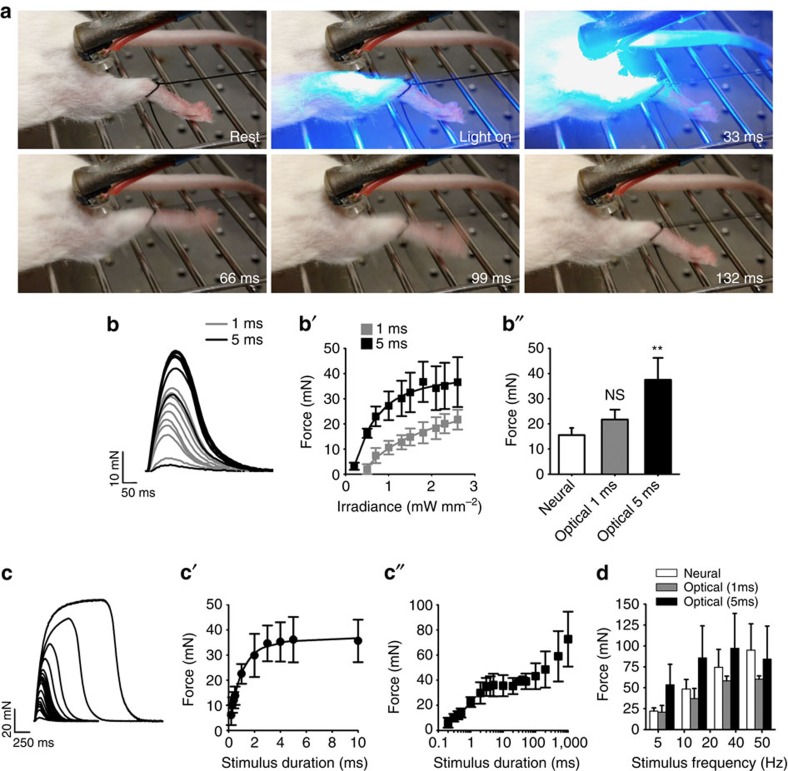
Optical stimulation modulates force by varying light intensity, duration and pulse frequency. (**a**) Hindlimb muscle contraction induced by blue light emitted from an LED positioned 1 cm away. Images represent five serial frames, 33, 66, 99 and 132 ms after the onset of a 50-ms light pulse. (**b**) Example of force gradations obtained by incrementally increasing light intensity using a 1- or 5-ms pulse of light. (**b**′) Quantification of force at different light intensities using a 1- or 5-ms pulse of light (2.6 mW mm^−2^). (**b**″) A 1-ms light pulse, at 2.6 mW mm^−2^, produces the same force as nerve-evoked twitch contraction, while a 5-ms pulse at the same light intensity produces significantly more force. (**c**) Force profiles generated while incrementally increasing light pulse duration from 0.2 ms to 1 s. (**c**′) Quantification of force generated using light pulses (2.6 mW mm^−2^) varying in duration from 0.2 to 10 ms. (**c**″) Quantification of force generated using light pulses (2.6 mW mm^−2^) varying in duration from 0.2 to 1,000 ms. Note that stimulus duration is plotted on a logarithmic scale. (**d**) Tetanized force can be graded by varying the frequency of neural or optical stimulation. Data are means±s.d. (*n*=4 per group). Statistical significance was determined using a one-way ANOVA followed by Dunnett's post-test; **P<0.01.

To ascertain whether muscles with this pattern of ChR2 expression contract when illuminated with light, we anaesthetized Sim1-Ai32 mice and pulsed the triceps surae muscles through the skin with blue light generated by a light-emitting diode (LED; 470 nm, 2.6 mW mm^−2^, 50 ms light pulse duration) positioned immediately above the dorsal shank musculature (Fig. 2a, top left panel). The time-lapse images show a typical example where the illuminated muscles contracted producing an ankle extensor response <66 ms after the LED was turned on (Supplementary Movie 1). To quantitatively measure contractile responses initiated by light, we conducted soleus muscle force recordings *ex vivo* using the same LED and compared those values with forces evoked by neural stimulation using a nerve suction electrode. Twitch forces progressively increased in response to increasing light intensities (Fig. 2b,b′) resulting in values best fitted to a four-parameter logistic curve (for 1-ms pulses; *r*^2^=0.991 with an effector concentration for half-maximum response (EC_50_)=1.94 mW mm^−2^, for 5-ms pulses *r*^2^=0.996 with an EC_50_=0.529 mW mm^−2^). While greater forces were produced using 5-ms light pulses (Fig. 2b″), we found force gradations were better controlled using 1-ms pulses, particularly for values <20 mN (Fig. 2b,b′). Interestingly, a single 1-ms light pulse of 2.6 mW mm^−2^ produced a force comparable to stimulating the nerve with a single electrical pulse (Fig. 2b″) indicating that, under these conditions, optical stimulation can be as efficient as neural stimulation. Twitches evoked by 5-ms light pulses, on the other hand, were stronger than those produced by neural stimulation (Fig. 2b″). This latter result suggests that contractile force can be graded by varying light pulse duration as well as light intensity. To examine this in more detail, we illuminated the soleus muscle with light pulses (2.6 mW mm^−2^) varying in duration from 0.2 to 1,000 ms. Remarkably, not only did contractile responses increase with longer pulse durations, there was no evidence of sag while the light was on (Fig. 2c). Twitch force values evoked with light pulses ranging from 0.2 to 10 ms were well fitted to a four-parameter logistic curve (*r*^2^=0.99 with an EC_50_=0.84 ms) (Fig. 2c′). Furthermore, forces plateaued with pulses ranging from 10 and 100 ms before increasing further with light pulses exceeding 100 ms (Fig. 2c″).

In mammals, contractile force is normally graded by recruiting progressively larger motor units^19^ and by modulating their firing frequency^20^. To ascertain whether stimulating muscles with varying frequencies of light pulses can similarly grade force, we illuminated the soleus muscle with 1- and 5-ms light pulses (2.6 mW mm^−2^) at 5–50 Hz for 500 ms. We then compared these values with those evoked by electrical nerve stimulation at the same frequencies. We found that tetanized force can be graded by varying the frequency of optical stimulation and that neural and optical stimulation generated comparable forces (Fig. 2d). Finally, to examine whether optically stimulated muscles have similar fatigability dynamics as neurally activated muscles, we compared forces evoked by neural and light activation during a 2-min fatigue test (see Methods for details). Under these conditions, we found that whole-muscle force fatigued more rapidly when stimulated optically compared with neural stimulation (Supplementary Fig. 1). Taken together, these results show that finely controlled light pulses efficiently produce contractile forces that can be graded by (1) increasing light intensity, (2) changing light pulse duration and (3) flashing light at varying frequencies.

### Light induces contractions independent of AChR activation

**Figure 3:**
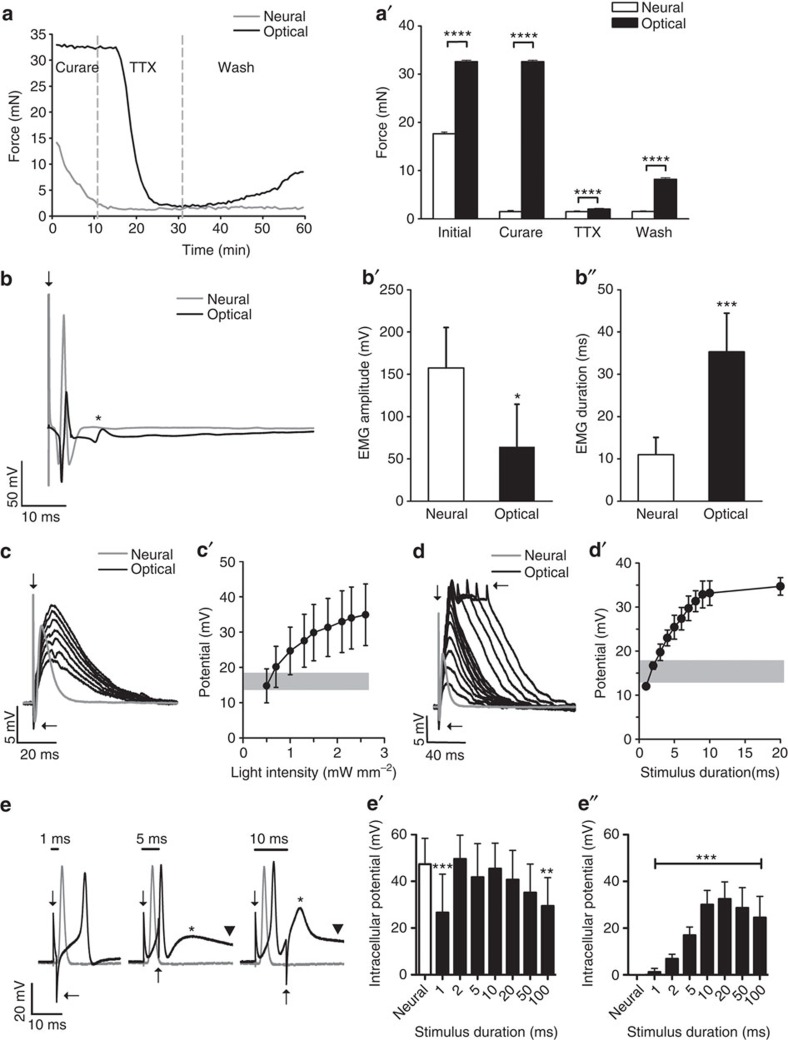
Muscle properties during light-induced contractions. (**a**) Sequential bath application of curare (10 μM) and TTX (500 nM) to soleus muscles shows that nerve-evoked contractions were blocked with curare while contractions induced by 5 ms light pulses were not. TTX, however, blocked light-evoked contractions. Partial recovery of light-evoked force occurred after a 30-min wash out. (**a**′) Quantification of nerve- and light-evoked force, before and after bath application of drugs. While light-evoked contractions were markedly attenuated in the presence of TTX, they remained minimally present (15 events averaged per condition). (**b**) Examples of EMGs evoked through nerve stimulation (grey trace), or with a 2.6-mW mm^−2^, 5-ms light pulse (black trace). The asterisk represents a second depolarization after light stimulation. (**b**′) Quantification of EMG amplitude and duration (**b**″) using nerve stimulation or a 2.6-mW mm^−2^, 5-ms light pulse (*n*=5). Note light stimulation produces EMGs that are smaller in amplitude, but longer in duration, compared with nerve stimulation. (**c**) Examples of muscle potentials recorded from soleus myofibres using nerve stimulation (grey trace) or with increasing light intensity (5 ms pulse, black traces). (**c**′) Quantification of evoked muscle potentials at increasing optically intensities (*n*=10). Shaded area shows range in EPPs recorded at the NMJ upon nerve stimulation. (**d**) Examples of muscle potentials recorded from myofibres using light pulses of varying lengths (1.0 mW mm^−2^ light intensity). (**d**′) Quantification of evoked muscle potentials recorded at varying light pulse durations (*n*=6). (**e**) Example of nerve- and light-evoked muscle depolarizations in the presence of myosin inhibitors. Note the second smaller response (asterisks) and sustained depolarization (arrowheads) with light pulses ⩾5 ms. Arrows represent stimulus artefacts when the LED was turned on and off. (**e**′,**e**″) Quantification of the first (**e**′) and second (**e**″) evoked potential recorded upon nerve stimulation (grey traces) and at varying light pulse durations (black traces) in the presence of myosin inhibitors (*n*=12). Bars and symbols indicate mean±s.d. Statistical significance as follows: in **a**, by two-way ANOVA and Bonferroni post-test; in **b**, by Student's *t*-test; in **e**, by one-way ANOVA followed by Dunnett's post-test **P<0.01, ***P<0.001, ****P<0.0001.

To elucidate how light pulses induce EC coupling in Sim1-Ai32 skeletal muscle fibres, we compared contractile forces elicited upon nerve stimulation with those induced by optical stimulation (2.6 mW mm^−2^, 5 ms pulse) while sequentially applying d-tubocurarine (curare; 10 μM) and tetrodotoxin (TTX; 500 nM) to soleus muscle/nerve preparations (Fig. 3a). As expected, nerve stimulation failed to induce contractions shortly after bath application of curare because of acetylcholine receptors (AChRs) blockade at the neuromuscular junctions (Fig. 3a,a′). In contrast, contractions induced by light pulses were not attenuated by curare indicating that ChR2 activation induces EC coupling downstream of synaptic transmission. Interestingly, light pulse-induced contractions were reduced by 97% when TTX was added to the bath (Fig. 3a,a′). These results suggest that light stimulation activates voltage-sensitive sodium channels causing action potentials to be generated within the sarcolemma and/or t-tubules before activating the release of Ca^2+^ from the sarcoplasmic reticulum. However, because some force was still produced by light pulses in the presence of TTX (Fig. 3a′; 1 mN, or 3% of initial force), these results also indicate that ChR2 activation alone can cause contraction. This likely occurs because the ChR2 current is either large enough to initiate the release of some Ca^2+^ from the sarcoplasmic reticulum or because Ca^2+^ entered the cell through the channel itself^9^.

### Depolarization increases with light intensity and duration

To examine electrical properties of optically stimulated muscles (2.6 mW mm^−2^, 5 ms pulses), we recorded electromyogram (EMG) activity using surface electrodes and compared the magnitude and duration of the signal with those produced by maximum, single-pulse nerve stimulation (0.2 ms pulse width). Interestingly, the peak-to-peak amplitude of the EMGs recorded from optically stimulated muscles was significantly less than those measured after nerve stimulation (Fig. 3b,b′). Because EMG amplitude is proportional to the number of synchronously active muscle fibres^21^,^22^, one could conclude that optical stimulation depolarizes fewer myofibres than nerve stimulation. However, we also noticed that the duration of the EMGs measured from optically stimulated muscles was significantly longer than those evoked by nerve stimulation (Fig. 3b,b″). EMG amplitude wanes if myofibres depolarize asynchronously, because the positive voltage from one fibre occurs together with the negative voltage of another^22^. Thus, in contrast to nerve stimulation, where muscle fibres depolarize relatively synchronously, optical stimulation depolarizes myofibres closest to the light first resulting in asynchronous activation and leading to smaller, but longer-lasting EMG signals. In addition, we also noted a second deflection in the EMG when the muscles were light activated with pulses ⩾5 ms (Fig. 3b, asterisk). This second deflection has the same time course as the second depolarization recorded from single-muscle fibres during a 5-ms light pulse (Fig. 3e). This second depolarization may therefore account for the second deflection in the EMG and, if so, prolongs its duration.

To examine electrical properties of individual myofibres, we used intracellular electrodes to record muscle potentials in response to nerve and optical stimulations. To prevent muscle contraction, 1 μM μ-conotoxin GIIIB (a muscle-specific voltage-gated Na_v_1.4 channel blocker) was added to the perfusion. Under these conditions, nerve stimulation produced large end-plate potentials (EPPs) that were similar in amplitude (∼18 mV) (Fig. 3c,c′, shaded area). In contrast, optical stimulation (5 ms light pulses) produced graded muscle potentials that increased with light intensity (Fig. 3c,c′). In addition, the rise and fall times of the potentials were significantly slower than nerve-evoked EPPs (Fig. 3c). Muscle depolarization could also be graded by increasing the light pulse duration while maintaining light intensity at 1.0 mW mm^−2^ (Fig. 3d,d′). Under these conditions, maximum depolarization occurred using 10-ms light pulses. Pulses longer than 20 ms or intensities greater than 1.0 mW mm^−2^ caused muscle contractions presumably because the depolarizing current was large enough to activate EC coupling. These persistent depolarizing currents could also account for the sustained contractions observed when muscles were optically stimulated with long light pulses (for example, see Fig. 2c). Contractions were then blocked by myosin inhibitors, 50 μM BTS (*N*-benzyl-p-toluene sulphonamide)^23^ and 10 μM blebbistatin^24^,^25^,^26^, applied simultaneously to the perfusion before intracellular potential recording of neural and optical stimulations (Fig. 3e). Under these conditions, light pulses longer than 1 ms consistently generated an action potential of identical amplitude to the neural-induced action potential (Fig. 3e,e′). As expected from the slower kinetics of ChR2, the optically generated action potentials were 4±1 ms delayed after the neural-induced action potentials. Interestingly, a second smaller depolarization (Fig. 3e, asterisks) was seen with stimuli greater than 1 ms that increased in amplitude with increasing light intensity (Fig. 3e″). In addition, a plateau in the depolarization occurred with light pulses ⩾20 ms that lasted until the light was turned off (Fig. 3e, arrowhead). Taking together, these results show that optical stimulation depolarizes myofibres asynchronously, induces EC coupling downstream of AChR activation, generates a second depolarization following the initial action potential and maintains prolong tetanic contractions by sustaining persistent muscle-depolarizing currents.

### Daily light pulse stimulation attenuates denervation atrophy

**Figure 4:**
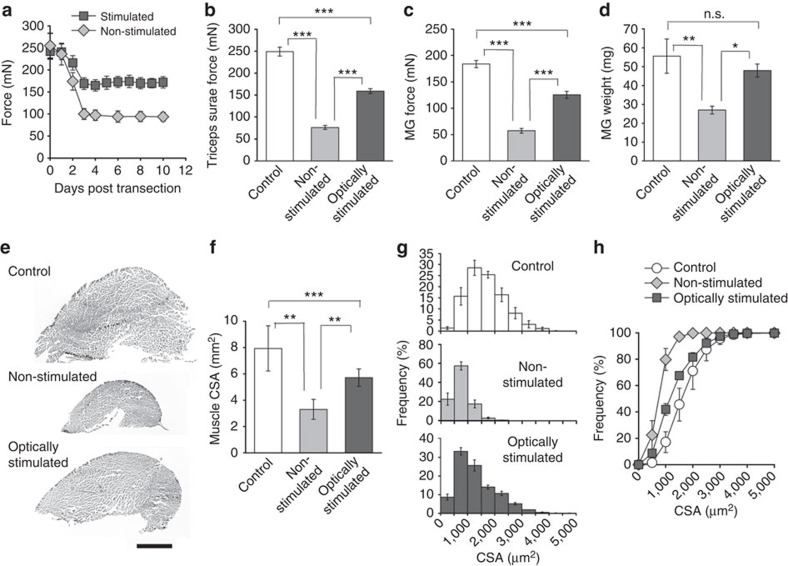
Daily transcutaneous light stimulation attenuates denervation atrophy and improves contractile force. (**a**) Average forces produced by optically stimulated and non-stimulated, denervated muscles recorded over 10 days following sciatic nerve transection. (**b**) Average twitch force recorded from contralateral (control), non-stimulated and optically stimulated triceps surae muscles 10 days after nerve transection. (**c**) Average light-induced twitch force and (**d**) weight wet of control, non-stimulated and optically stimulated MG muscles 10 days after nerve transection. (**e**) Whole-muscle cross-sections of control, non-stimulated and optically stimulated MG muscles stained for haematoxylin and eosin. (**f**) Mean muscle cross-sectional areas of the three muscle groups. (**g**) Frequency histograms comparing the cross-sectional area of myofibres in control, non-stimulated and optically stimulated MG muscles. (**h**) Cumulative frequency showing the shift to smaller muscle fibres in the non-stimulated muscles compared with optically stimulated and control muscles. Data indicate mean±s.d. (*n*=6 animals per group). Statistical significance was determined with one-way ANOVA followed by a post-test using the Holm–Sidak method; *P<0.05, **P<0.01,***P<0.001. CSA, cross-sectional area; NS, not significant. Scale bar, 1 mm (**e**).

To investigate whether channel rhodopsin expression in muscle could be used to restore function to permanently denervated muscles, we cut and ligated the sciatic nerve in Sim1-Ai32 mice. One cohort of animals (designated stimulated) received daily transcutaneous light stimulation (5 ms pulses at 20 Hz for 500 ms every 6 s for 1 h) to the triceps surae muscles for 10 days, starting immediately after the nerve was transected^27^. Another cohort was not stimulated with light (designated non-stimulated). EMG recordings from denervated medial gastrocnemius (MG) muscles in freely moving Sim1::A132 mice showed no signs of muscle activation, indicating that ambient light did not cause muscle contractions. The contralateral muscles served as unoperated controls. Mice were anaesthetized at regular intervals, starting immediately before nerve transection, to measure twitch force produced at the ankle joint upon transcutaneous light stimulation (2.6 mW mm^−2^ with a 5-ms pulse width). Figure 4a shows average daily forces produced by the optically stimulated and non-stimulated denervated muscles, recorded over a 10-day period. In both cases, force produced at the ankle by the triceps surae muscles decreased significantly from pre-operative levels. However, the attenuation in force was significantly less for muscles receiving daily optical stimulation such that the force of optically stimulated muscles plateaued at ∼70% pre-operative levels, while the non-stimulated muscles plateaued at ∼40% (Fig. 4a,b). At 10 days after nerve transaction, the MG muscles were isolated *ex vivo* and their tendons attached to a force transducer. Light-activated contractions generated from the optically stimulated and non-stimulated muscles were then compared with the contractile forces generated by optically stimulating the contralateral control MG muscle. Figure 4c shows that, while the contractile force of MG muscles receiving daily optical stimulation was significantly less than control values (∼70%), they were also significantly stronger than non-stimulated muscles. This difference in force was reflected in their wet weights (Fig. 4d) and overall size (Fig. 4e). The cross-sectional areas of the optically stimulated muscles were significantly smaller than control muscles, but significantly larger than non-stimulated muscles (Fig. 4f). As expected, non-stimulated denervated MG myofibres were also significantly smaller than fibres in control and optically stimulated muscles (Fig. 4g,h), indicating that denervation atrophy was most pronounced in this experimental group. Taken together, these results indicate that daily transcutaneous light stimulation can significantly decrease the effects of denervation in muscles expressing ChR2.

## Discussion

In this study, we show that ChR2 is abundantly expressed within the sarcolemma and T-tubules of skeletal muscle fibres in Sim1-Ai32 mice. This expression pattern allows for efficient control of muscle contraction using light pulses from a 470-nm LED, where maximal force obtained by optical stimulation was comparable to direct nerve stimulation. Furthermore, muscle force could be graded in a controlled manner by increasing light pulse intensity, duration or by modulating their frequency. Muscle fibres depolarized in a graded manner relative to light stimulation until reaching action potential threshold and for longer pulse durations remained depolarized for the duration of the light pulse. Although the gradation in force was likely due to an increase in the number of muscle fibres activated and the contractile strength of each fibre, it is not the same as neurally evoked activation in force, which normally occurs according to the size principle^19^. Finally, we found that muscle fibre atrophy was significantly attenuated when the denervated muscles were optically stimulated through the skin for 1 h a day. This decrease in atrophy resulted in significantly greater contractile forces compared with chronically denervated muscles. Together, this proof-of-principle study shows that stimulation of ChR2-expressing myofibres with light can restore function to denervated muscles and reduce pathophysiological changes associated with injury. Furthermore, it extends on recent work by Sasse *et al*.^28^, who showed optical control of cardiac muscle, as well as the reports by Delp *et al*. illustrating muscle activation via optical stimulation of the sciatic nerve^29^.

To date, restoration of function to paralysed muscles typically involves electrical stimulation. FES has been studied and used to restore function to upper and lower extremities, the diaphragm, bladder and bowels^3^. Electrical impulses are typically applied to electrodes near, or around, the peripheral nerve causing motor axon depolarization and muscle contraction. This approach, however, is not useful if the target muscles are completely denervated, as occurs after SCIs^30^,^31^, motor neuron diseases such as amyotrophic lateral sclerosis^32^ and severe peripheral nerve injuries^33^, such as occurs after a brachial plexus avulsion. It is not useful because denervated muscles require substantially more current to induce contractions compared with neural stimulation. High currents promote a multitude of problems including electrode corrosion, tissue damage, discomfort and unwanted stimulus spread to neighbouring muscles^34^.

To circumvent these issues, we^6^,^7^ and others^5^,^8^ restored innervation and function to denervated muscles by transplanting embryonic motor neurons, or motor neurons derived from pluripotent cells, into the peripheral nerve near the nerve–muscle entry point. Electrically stimulating the transplanted neurons generated between 12 and 40% of the original contractile force. While promising, this approach has many technical and biological obstacles that may ultimately circumvent its use clinically. Foremost among these is the justifiable concern that teratomas will form^35^. Although the risk of teratoma formation can be reduced experimentally by removing unwanted pluripotent cells before transplantation using small molecules^36^, immunodepletion^37^, genetic selection^38^ or by introducing a cytotoxic antibody^39^, in practical terms it may be very difficult to guarantee their complete absence to a regulatory agency. Furthermore, it is uncertain whether progenitor or partially differentiated cells remaining in the transplant will someday become a health risk to the transplant recipient during their life span^40^.

Transplantation of motor neurons to restore muscle function after injury will likely occur only after endogenous recovery is complete, a process that can take several months in humans^41^. This reality further obstructs the use of motor neuron transplantation, because motor axon regeneration and muscle fibre reinnervation is very poor when axotomy and/or muscle denervation is prolonged^11^,^12^,^41^,^42^,^43^. Thus, while restorative transplantation therapies using motor neurons derived from pluripotent cells hold promise, practical limitations may ultimately hinder clinical application of this technology.

Restoration of muscle function by direct optical activation of myofibres abates risks of teratoma formation and bypasses the need to restore function by regenerating axons through an inhospitable environment. Skeletal muscles are amenable to different delivery mechanisms for gene delivery such as viral transduction. Indeed, gene transfer into adult muscle has recently been shown to express the ChR2/H134R, resulting in functional muscle contraction under light stimulation^44^. While our study is largely a proof of principle using transgenic animals, it is possible that, in combination with gene transfer technology, this strategy may become clinically useful for restoring function to specific muscles permanently denervated by injury or disease. However, the success of this strategy will still be highly dependent on viral delivery method, sustained expression of ChR2 in infected fibres and kinetics of engineered ChR2. Nonetheless, restoring even a small amount of function will improve independence and have a profoundly positive effect on the adverse psychological, social and economic factors associated with denervation injuries, ranging from peripheral nerve injuries^33^ to SCIs^1^.

## Methods

### Mouse strain

*Sim1*^*cre/+*^ mice (CD1/C57Bl/6J mixed background), described elsewhere^45^, were crossed with Ai32 mice (C57Bl/6J background) obtained from the Jackson Laboratory (strain B6; 129S-*Gt(ROSA)26Sor*^tm32(CAG-COP4*H134R/EYFP)Hze^/J, stock number 012569) to generate Sim1-Ai32 mice. These mice express a modified ChR2/EYFP fusion protein where the opsin protein harbours a gain-of-function H134R substitution permitting to generate larger photocurrents^16^ under blue light (450–490 nm) stimulation. *Sim1*^*cre/+*^ animals were genotyped for ChR2 and the Cre-recombinase sequences using the following primers (5′–3′): forward ACATGGTCCTGCTGGAGTTC , reverse GGCATTAAAGCAGCGTATCC and forward CCGGTGAACGTGCAAAACAGGCTCTA , reverse CTTCCAGGGCGCGAGTTGATAGC , respectively.

### *In vitro* soleus muscle isometric tension

Adult (6–8 weeks) female Sim1-Ai32 mice were used throughout this study. All procedures were approved by the ethics committee at Dalhousie University and followed the Canadian Tri-Council guidelines for laboratory animals. Under isoflurane anaesthesia, animals were killed by cervical dislocation and the left soleus muscle (*n*=5) was quickly dissected out in ice-cold oxygenated (5% CO_2_/95% O_2_) Tyrode's solution (125 mM NaCl, 24 mM NaHCO_3_, 5.37 mM KCl, 1 mM MgCl_2_, 1.8 mM CaCl_2_ and 5% dextrose) before transferring to a Sylgard-coated (Dow Corning) recording chamber perfused with oxygenated Tyrode's solution at room temperature. The distal tendon was attached to a force transducer (FT03, Grass Technologies) with a silk tie to measure isometric contraction. A glass suction electrode (BF120-90-10, WPI) was used to deliver electrical stimulation to the nerve via an S88 stimulator (Grass Technologies). The stimulus was isolated from ground by a stimulus isolation unit (PSU6, Grass Technologies) delivering a monophasic supramaximal stimulus, defined as 1.5 times the stimulus for maximal twitch force (∼100 μA), with a pulse duration of 0.2 ms. EMG was recorded with a polyethylene suction electrode (PE-190, Clay Adams) positioned on the soleus muscle belly. EMGs were amplified with a 10 × pre-amplifier (4001 differential headstage, Dagan Corporation) and further 10 × amplified and bandwidth filtered between of 3 Hz and 3 kHz (EX4-400, Dagan Corporation). EMG amplitude was measured as the maximum excursion between the positive and negative phases of the waveform (as defined by isoelectric crossings), while EMG duration was quantified as the time between the onset and offset of the first and second phase of EMG potential. Force and EMG responses were acquired at 10 kHz using a Digidata 1322AA/D board (Axon Instruments) and Axoscope 9.2 software (Axon Instruments). Optical stimulation was performed with a blue (470 nm) Rebel Star O Drop-in LED from Luxeon Star (Quadica Development Inc.) positioned 1 cm above the muscle. The LED current was controlled with an a.c./d.c. converter connected to a solid-state relay triggered with a S88 stimulator (Grass Technologies). The LED irradiance was measure with a photodetector amplifier (PDA1, World Precision Instrument) connected to a silicone planar photodiode (VISD, Word Precision Instruments) positioned at 1 cm distance from the light source. Fatiguability of whole-muscle force was compared between nerve and optical stimulation using 40-Hz electrical and light pulses (5 ms), respectively, that were sustained for 350 ms, repeated every second for 2 min. Fatigue index was quantified by dividing force values at 1 and 2 min by the initial tetanic force at the onset of the fatigue test.

### Drug treatments

Muscle contractions were determined in the presence of the following drugs: 10 μM d-tubocurarine (Sigma T2379), 500 nM TTX (Abcam ab120055), 1 μM μ-conotoxin GIIIB (Alomone Labs C-270), 50 μM 4-methyl-*N*-(phenylmethyl) benzene sulfonamide (BTS) (Tocris Bioscience no 1870) and 10 μM blebbistatin (Sigma B0560).

### Intracellular myofibre electrophysiology

To prevent muscle contractions, intracellular muscle fibre recordings were performed in well-oxygenated Tyrode's solution in the presence of μ-conotoxin (*n*=10) or with BTS and blebbistatin (*n*=12). Nerve- and light-evoked potentials were generated as described above. Sharp glass electrodes (BF100-50-10; World Precision Instruments) with resistances between 20 and 40 MΩ were filled with 3 M KCl and connected to an intracellular amplifier (Duo 773 Electrometer, World Precision Instruments). Signals were amplified and bandpass filtered between 1 Hz to 10 kHz and digitized as described above. Recordings were discarded if the initial membrane resting potential (−65 to −90 mV) changed by more than 10% of its original value.

### Soleus muscle immunofluorescence and imaging

Soleus muscles were isolated, pinned on a cork, embedded in OCT (VWR) and flash frozen in dry ice-cooled isopentane (Sigma). For immunofluorescence, 20 μm muscle sections were cut on a cryostat (Leica) and mounted on microscope slides. Slides were fixed for 20 min in 4% paraformaldehyde and washed in PBS before incubation with antibodies in 0.3% Triton-X (Sigma) in PBS plus 10% goat serum. Incubation with primary antibodies was performed overnight, whereas secondary antibody incubation was done over 2 h. Antibodies consisted of: rabbit anti-GFP IgG (Millipore AB3080, 1:1,000), mouse anti-dihydropyridine receptor α2-subunit (Sigma D218, 1:500) goat anti-rabbit IgG conjugated to Alexa Fluor 488 (Life Technologies A-11008, 1:500) and goat anti-mouse conjugated to Alexa Fluor 546 (Life Technologies A-21045, 1:500). Slides were mounted with fluoromount-G medium (Fischer Scientific, 0B100-01). For sarcolemma and T-tubule labelling, soleus muscles were isolated after trans-cardiac perfusion with 4% paraformaldehyde and post-fixed for 2 h at 4 °C, subsequently washed in PBS and incubated for 1 week in a 1 mg ml^−1^ DiI solution (Molecular Probes) in PBS at 4 °C (ref. 46). Stained muscles were washed with PBS, flash frozen and sectioned as described above. Images were acquired with a Zeiss LSM 710 confocal scanning microscope equipped with Zen 2009 software (Zeiss).

### Hindlimb denervation and stimulation

Sim1-Ai32 mice were anaesthetized with isoflurane and the right sciatic nerve was transected under aseptic conditions. Both proximal and distal ends of the sciatic nerve stump were ligated with 4-0 sutures to prevent regeneration. Hair was removed from the hindlimbs using standard mouse shears. The triceps surae muscles were stimulated transcutaneously using a blue (470 nm) Rebel Star LED positioned immediately above the dorsal shank. The mice were randomized into a stimulated (*n*=4) and non-stimulated group (*n*=3). The contralateral side served as a non-operated control group. Light pulse stimulation began immediately after the sciatic nerve was transected. The daily stimulation protocol consisted of a 5-ms light pulse at 20 Hz for 500 ms every 6 s for 1 h (a total of 600 tetanic contractions per day), a protocol shown to increase muscle force and weight of denervated rat muscle^27^. At regular intervals, the mice were anaesthetized with isoflurane, the right ankle and knee stabilized and the foot connected to a FT03 force transducer to measure torque at the ankle, generated by a 5-ms light pulse (2.6 mW mm^−2^). After 10 days of daily stimulation, the mice were anaesthetized, the knee firmly fixed in place with a stereotactic frame (Kopf) and the calcaneus was connected to a force transducer to record light-activated contractions. Great care was taken not to disturb the blood supply to the tricep surae muscles. After force recordings, the MG muscles were excised and flash frozen as per above. Cross-sections (10 μm) were cut from the mid region of the muscle before staining with haematoxylin and eosin (Sigma). Whole-muscle and individual myofibre cross-sectional areas were quantified using Image J (NIH).

### Statistical analysis

Statistics were performed with Prism5 (GraphPad) and Sigmaplot. Student's *t*-tests were done when comparing between only two groups, otherwise one- or two-way analysis of variance was performed for multiple groups followed by a Dunnett's or a Holm–Sidak post-test with the *P* value significance set at 0.05. Sample size of most experiments was chosen empirically following previous experience in the assessment of experimental variability with *n* always ⩾3 animals. *P*<0.05 was considered statistically significant.

## Additional information

**How to cite this article:** Magown, P. *et al*. Direct optical activation of skeletal muscle fibres efficiently controls muscle contraction and attenuates denervation atrophy. *Nat. Commun.* 6:8506 doi: 10.1038/ncomms9506 (2015).

## Supplementary Material

Supplementary Figure Supplementary Figure 1

Supplementary Movie 1 Transcutaneous optical stimulation efficiently controls contraction of mouse calf muscle expressing ChR
