# Supplementary material for: Direct optical activation of skeletal muscle fibres efficiently controls muscle contraction and attenuates denervation atrophy
Source: Nat Commun. 2015 Oct 13;6:8506. doi: 10.1038/ncomms9506 (PMC4633712; doi:10.1038/ncomms9506)
Supplement: Supplementary Figure — 1 [file ncomms9506-s1.pdf]

Supplementary Figure 1.

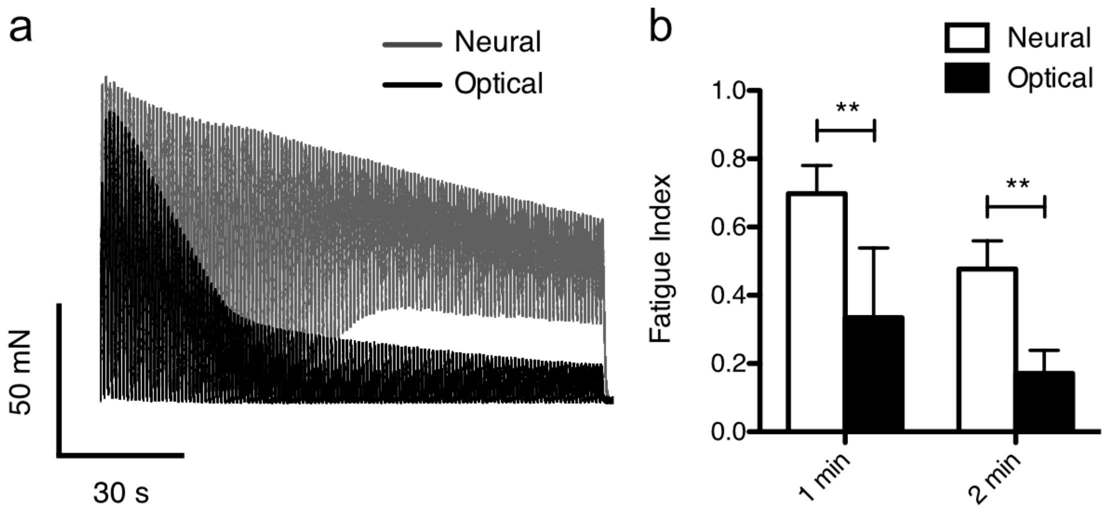

Fatigue test performed using neural or optical stimulation. Stimulation was performed at 40 Hz stimulation sustained over 350 ms and repeated every second for 2 minutes. Parameters for stimulation were those of twitch stimuli as described in methods. (**a**) Representative force curves show a more rapid fatigue of optical stimulation compared to neural stimulation. (**b**) Bar graph of fatigue index under neural and optical stimulation at one and two minutes (n=6). Data represented as means ± s.d. Statistical significance by two-way ANOVA with Bonferroni multiple comparisons. ** $p<0.01$
